# Top Tips for Designing and Managing a Public Engagement Laboratory

**DOI:** 10.1021/acs.jchemed.3c01053

**Published:** 2024-02-27

**Authors:** Martin McHugh, Ebru Eren, Genco Guralp, Krishna Hari, Sarah Guerin, Sarah Hayes, Colm O’Hehir, Eamonn O’Sullivan, Alida Zauers, Caitriona Tyndall

**Affiliations:** † SSPC, the SFI Centre for Pharmaceuticals, Bernal Institute, University of Limerick, Limerick V94 T9PX, Ireland; ‡ IPIC, the SFI Centre for Photonics, Tyndall National Institute, University College Cork, Cork T12 CY82, Ireland; § Tyndall National Institute, University College Cork, Cork T12 R5CP, Ireland

**Keywords:** Public Understanding/Outreach, General Public, Lab Space

## Abstract

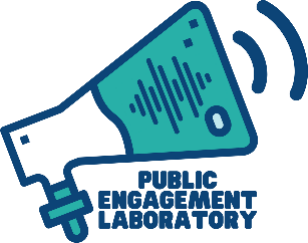

Public engagement with science is a core facet of the broader science ecosystem, in particular the science research and science education sectors. In this article we demarcate the benefits of dedicated laboratories along with practitioner advice pertaining to the design and running of a public engagement learning environment. A practicing public engagement laboratory and one that is currently being developed are used as illustrative cases to provide real-world insights to public engagement practitioners.

## Introduction

Public engagement refers to actively working with the public in the area of Science, Technology, Engineering, and Mathematics (STEM).^1,2^ The space is host to a taxonomy of approaches and terminologies; inclusive in this is “outreach”. Not limited to the typical view of classroom visits by scientists,^3^ public engagement approaches are diverse. Nonformal science education includes collaborations with museums and zoos.^4^ Science can also be brought to historical museums^5^ or public libraries.^6^ to promote cross-subject linkages and equitable access for members of the public. Indeed, access is the motivation behind mobile laboratories^7,8^ that can be transported to schools and public events and transformed with expanding pods into classrooms.^9^ Dual purpose undergraduate laboratories can support local schools,^10^ after school clubs,^11^ summer schools,^12^ and field trips to scientific institutions.^11,13^ A service learning program with local communities can also instill a sense of responsible research and innovation along with the development of transferable skills in graduate and university staff, creating a symbiotic relationship.^14^ The goodwill generated by these projects has led to longevity with well-established and varied public engagement offerings being designed and delivered by universities for the past 20 years.^6,15^ Prolonged engagement has led to the development of public engagement where cocreation and community engaged research^16,17^ are paramount to informal and nonformal public engagement with science.^18^

While the above approaches to public engagement have a variety of goals, in general, it is undertaken to provide thought provoking and inspirational science that can help change participants’ perspectives and attitudes.^4,14,19^

Science has fallen afoul to persistent public perceptions that it is a difficult subject area albeit with high societal importance.^8^ The global scientific literature is abounded with references to the “leaky STEM pipeline” with students selectively moving away from science throughout their education,^20^ a phenomenon that is more evident among women^21^ and those from LGBTQ+ backgrounds.^22^ Yet, market competition is driving the requirement for students who pursue STEM along with the need for STEM literacy across the general population.^8,23,24^

It is with this background that public engagement forms a cornerstone of the science education landscape.^19^ Public engagement is a valuable supplement to formal education, and the role of scientists and graduate students in science education has been espoused by research and governmental organizations such as the NSF^25^ and the European Commission.^26^

From an Irish perspective, Science Foundation Ireland (SFI), a funding body equivalent to the National Science Foundation (NSF) in the United States, funds scientific research with an embedded expectation that public engagement will also be facilitated. This agenda has been strongly promoted since 2013 and now 16 SFI research centers (two of which form the authorship team of this paper) enable innovative discovery, collaboration, and opportunity in science, while placing public engagement at its core to future proof STEM. Central to this idea is the appointment of public engagement staff that are hosted in third level institutes throughout Ireland. Mandated to engage the public with STEM and the scientific research of the center, public engagement staff along with center researchers have delivered thousands of activities to the Irish public and beyond. Such activity includes the development of science workshops,^2^ professional development in science communication,^27^ and larger engaged research with communities.^28^

While the model has seen high adoption, the rapid proliferation of public engagement does pose challenges with regard to institutional support. A limiting factor to the quantity and more importantly quality of public engagement is infrastructure supported by professional staff.^4^ Third level institutes often lack public-facing spaces. This is particularly true for science where lab space and time is a valued commodity. However, requests to visit scientific institutions are commonplace. The logistics and skills required for small adult groups versus 100 children are vast with considerable administration burdens.^10^ Disruption, noise, and traffic are concerning if research is slowed or impaired.^10^ In addition, all visitors to third level scientific institutions represent a health and safety risk that requires moderation.^3^ We add to this and posit that a dedicated laboratory to host visitors and store equipment can complement the vast array of public engagement practices within third level institutes. Not only can these laboratories facilitate repeated visits, but they also act as a space for public engagement, creation and experimentation, preparation, and codesign.

While the idea of having a dedicated lab for public engagement is not new,^3^ with numerous dedicated laboratories in Great Britain, for example,^10^ there is a dearth of literature describing their design and implementation. With this backdrop, the following will demarcate a practitioner’s guide to permanent laboratory spaces for public engagement. Two illustrative Irish examples from an established public engagement laboratory (SSPC Centre) and one that is currently being designed (Tyndall National Institute and the IPIC Centre) will be explored while detailing aspects of design, implementation, and longevity.

## Needs Analysis

Whether a public engagement laboratory is to be incorporated into existing infrastructure or included in a new build, evidencing the need for a dedicated space is a key step. A needs analysis should entail theoretical and practical considerations grounded in the documentation of prior public engagement work along with future plans enabled by the lab space.

While the focus of a public engagement practitioner on educational initiatives will be on creating a stimulating learning environment that aids in cognitive development, critical thinking, and problem solving through scientific experiences and experiments, the focus of other university staff may be very different. Managers and Professors might be concerned with time expenditure (staff and room time), disruption (or lack thereof), public relations/marketing, and campus awareness, and these are all areas that must be considered, in particular their alignment with intuitional goals and strategy. It may be necessary to illustrate such impacts monetarily through efficiency savings. In addition, a predictive timetable for the lab to demonstrate that it will be fully utilized will need to be developed. The existing SSPC lab caters for approximately one visit per week from participants. However, it is also used for the preparation and storage of all public engagement equipment and materials along with group meetings and presentations. Another consideration is the role of the lab in the development of public engagement activity. Every aspect of workshops and lessons requires fleshing out and testing. A public engagement laboratory allows for better design and bespoke solutions to “messy” real world engagement with participants.

Finally, the lab should be positioned as a communal hub for your institute that can support other activities. The IPIC lab is designed with the guiding principles of “Welcoming, Engaging and Stimulating”. This approach, along with specific features of the lab, was codesigned with the wider institutional community. Aligned with this, the SSPC lab can be readily booked by anyone in the institution by contacting members of the public engagement team. To date the lab has been used for researcher training and filming/photography due to the space being branded and tidier than other laboratories in the institute that would normally require cleaning before any such activity.

## Lab Design and Features

A public lab space should be designed to enable experiential science. The “doing” of science is broadly underpinned by the benefits of active learning, and this should be reflected in the design of any public engagement laboratory. While most lab designs will consider the need for chemical storage, sinks, and fume hoods, there are other elements required in a public engagement laboratory.

**Figure 1:**
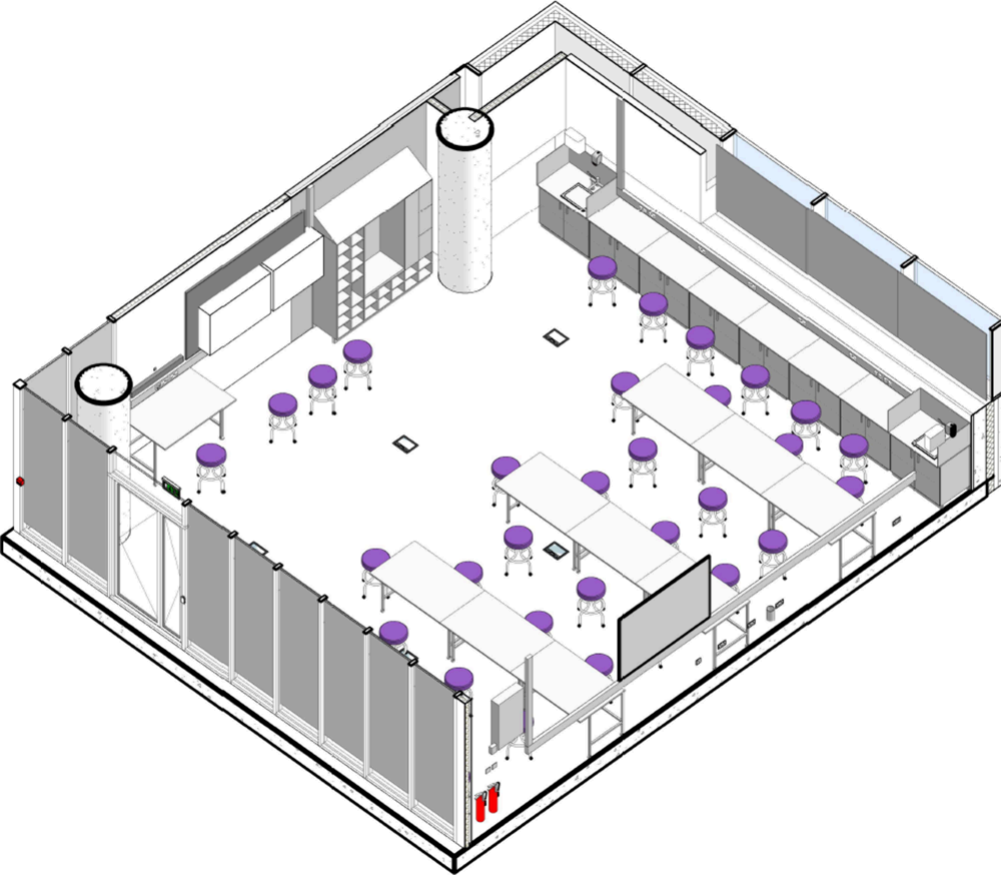
Isometric view of the planned IPIC public engagement laboratory.

The first step is creating a multimodal lab layout. Figure 1 displays an isometric view of the proposed IPIC lab in which all furniture is mobile allowing the space to be optimally configured based on the visiting participants and work to be conducted.

**Figure 2:**
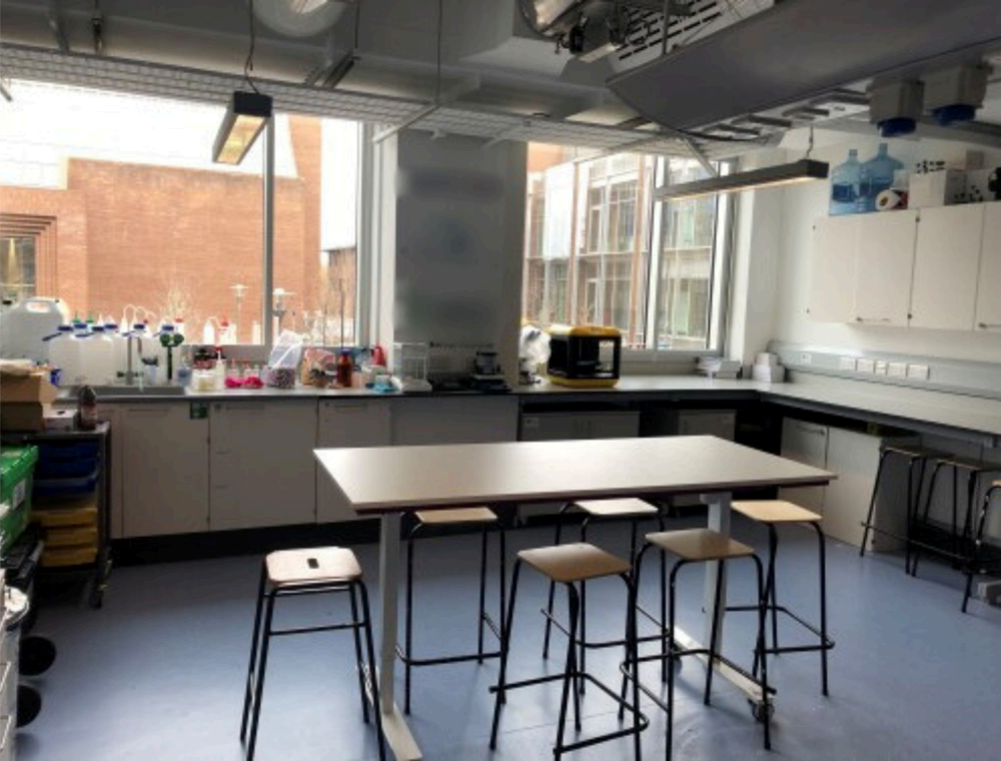
Picture of the SSPC public engagement laboratory with height adjustable and movable table and lockable wall storage.

The initial design must take into account the amount of people you can safely host in the lab, the differing scientific workshops that you plan to implement, and storage of said workshops along with space for new workshops and experiments. Moreover, the lab may host groups from young children to retirees, the space needs to be able to shift and change to your needs. An example of this is that both the SSPC and IPIC laboratories have height adjustable desks that are on wheels (Figure 2). Not only does this help ensure access and inclusion^10^ but the layout of the lab can be changed to help set up certain experiments or enable pedagogies such as group work. Moreover, the IPIC lab will have sinks at different heights to ensure accessibility.

Storage is also a key factor that can enable flexible learning spaces. A public engagement lab should host a range of storage options to support the quick deployment of any experiment. Ideally storage should be lockable as in SSPC we have found that lab visitors of all ages will open cabinets they are seated near. While a lock and key does not remove the participant’s curiosity, it does remove a potential health and safety risk for the public engagement practitioner. Furthermore, ample storage allows the lab to always look tidy, and this is important for the image of your host institution.

**Figure 3:**
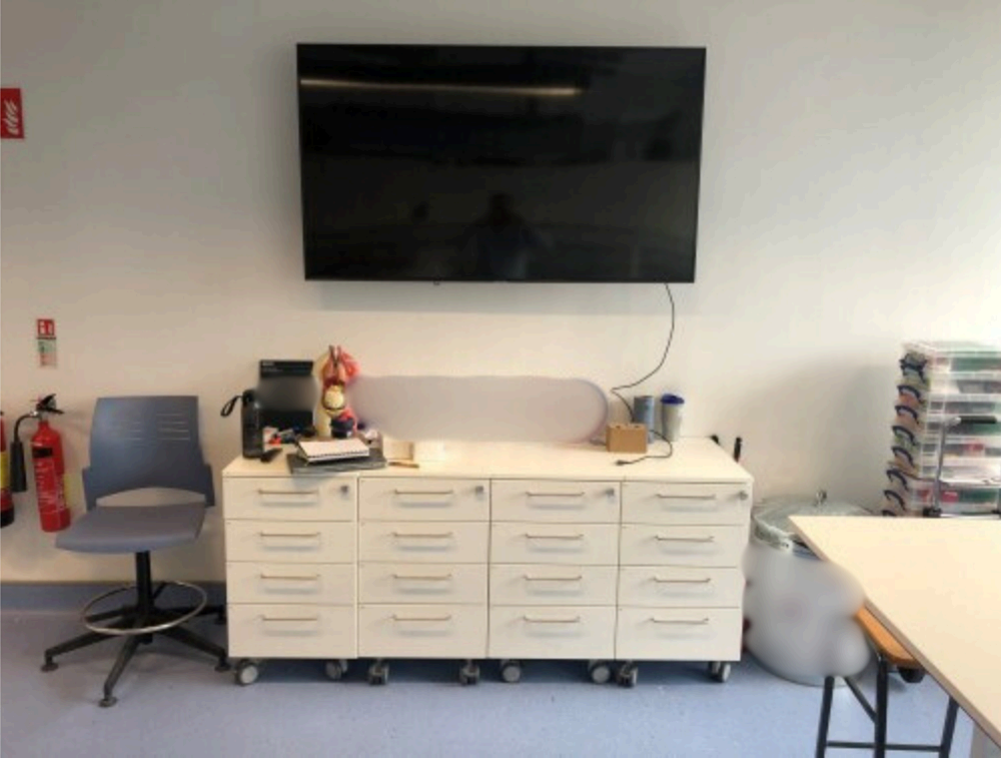
SSPC installed a 57-in. screen instead of a projector as this suited the layout of the lab. The screen is also not obstructed by light coming in from the windows.

**Figure 4:**
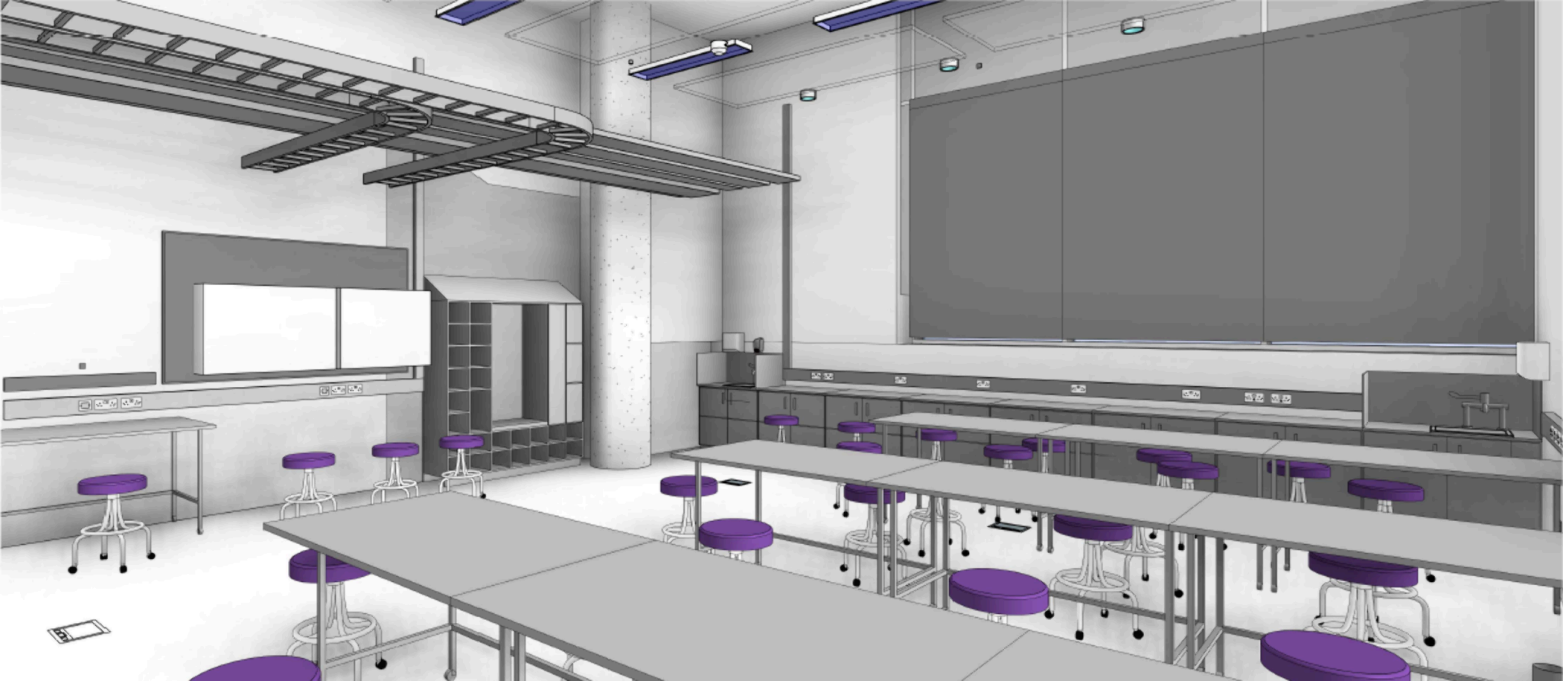
Illustration of the IPIC laboratory with windows on the right-hand side covered by blackout blinds.

Another consideration is windows, light, and orientation. If the sun shines into your lab, you may need to have blinds to ensure that participants can see the screen. With this in mind, does a screen, interactive whiteboard, or a projector work best in your space and where is it best placed in your lab (Figure 3)? For example, the IPIC lab will have two walls that are entirely made of glass, one facing a service road and the other facing a public exhibition space and café. The glass walls facing the public space will allow visitors to observe activities taking place in the lab. While this setup will provide for a visually stimulating and inviting environment, the focus of IPIC’s research on photonics and light-based experiments will be central to the activity in the lab. In this way, excess light shining through glass walls will be a hindrance to experimentation and that is why black-out blinds with multiple control points will be installed (Figure 4).

Essentially, the design of a public engagement laboratory is to predict and ameliorate potential snags when participants partake in public engagement. However, multiple issues arise in practice, and the following will demarcate SSPC’s journey of running a lab for the past 3 years, with a case study, and IPIC’s emergent learning from creating a new space from scratch.

## Implementation in Practice

A public engagement laboratory should run in the same way as any other lab in your institute. This includes items such as chemical storage, cleaning services, and lab booking. The key differentiator is that you will frequently have participants in your lab for whom it may be their first time on a campus, let alone in a lab, and a public engagement practitioner must adopt certain practices to ensure a safe and enjoyable experience.

With any group set to visit the lab, you should have good communication through phone or email with the head of the group or teacher. It is essential to have a list of all the people visiting on the day along with any additional information you may need such as emergency contact numbers and information on allergies. In the Irish context, most school visitors will be wearing a uniform; otherwise, a hat or sash can be used for quick identification. When communicating with a group of participants, clear instructions pertaining to building locations, meeting points, drop-off points, and parking are warranted. In SSPC, we are fortunate that our building is located beside the University bus stop. Therefore, we can send participants an image of the building and meet them in the foyer. Participants then follow us up a flight of stairs and through a corridor to the lab. There are elevators on site, and disabled access and restrooms are also present. The distance between the foyer and the lab is around 150 meters, and while this is a relatively short distance, participants have still managed to get lost, lose belongings, and disturb staff in offices through noise generation. Due to this, when we meet participants in the foyer, we give them a brief welcome along with some simple instructions about sticking together, holding doors open for the person behind them, taking care of their personal belongings and being sure not to disturb staff who are busy at work. With the above in mind, the IPIC lab will be built along a popular walking trail roughly 5 min from the city center. The lab is immediately visible through the visitor entrance and accessible via swipe access. An additional entrance for visitors who arrive via bus will be through the main entrance on the opposite side of the building.

### Health and Safety

Health and Safety is an immediate concern once participants are on the grounds of your institute. General lab health and safety rules around tying hair back, wearing closed shoes and long trousers, and use of lab coats and safety goggles need to be followed. From a broader context, you need to make sure campus security is aware of the visit along with health and safety staff belonging to your department and building. From an Irish perspective, public engagement staff also require national police clearance to work with people under the age of 18. If school children are visiting your lab, they need to be accompanied by their teacher at all times and your department should have a visitor procedure that outlines the ratio of teachers to students visiting. In addition, visitors may need to sign documentation acknowledging that they are aware of the risks of any potential activity and photo disclosure forms if appropriate.

**Figure 5:**
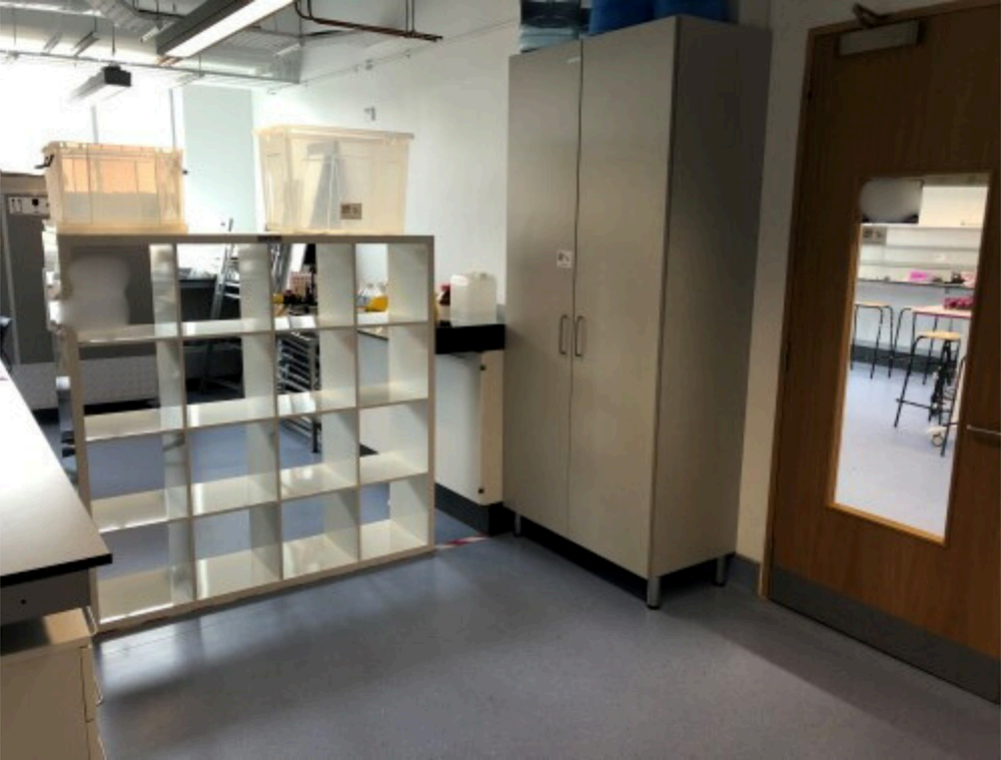
Holding area before entering the lab with storage for coats and bags. Participants can also receive instructions in the space and put on PPE.

**Figure 6:**
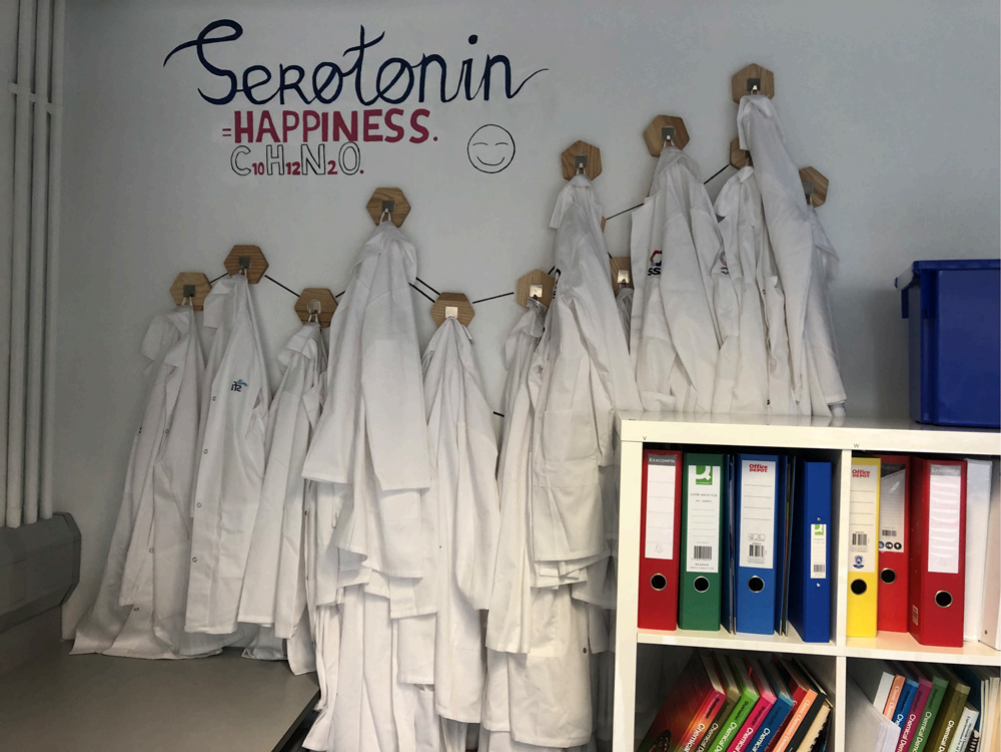
Children’s lab coats for participants aged 6–12 with artwork on the wall.

Just before entering the lab, SSPC has a “holding area” in which participants can leave their bags and jackets (Figure 5). This area can also be used to go through any safety instructions while donning personal protective equipment (PPE). It is important that participants do not wear cumbersome clothing under their lab coats. This poses a safety risk, and participants will complain about being too warm a few minutes into the workshop. In SSPC, we have a set of children’s lab coats that can be used for participants between the ages of 6–12 (Figure 6). Before entry into the lab, participants should be informed of where to sit and if they are they are working in groups. The IPIC lab will have storage with combination locks while lab coats will be stored in a dedicated cabinet akin to a working laboratory.

In terms of practice, any experiment taking place should have a risk assessment or chemical risk assessment as appropriate. In both the SSPC and IPIC laboratories, plastic is used over glass where possible, and all cabinets are lockable as curiosity can get the better of younger participants. In line with this, equipment should be distributed after an introduction to the workshop as participants will immediately begin to investigate any equipment that is within hands reach upon sitting down.

### Working with Your Institute

Longevity is a key issue if the lab is to have continued positive impacts with local participants, e.g., schools. To enable long-term impacts, the public engagement lab should seamlessly fit within the day to day running of your institute. Upon opening, you may need to present the idea behind the lab along with short- and long-term goals so that the wider community understands the work that will be taking place. This will help ensure that you maintain goodwill and the lab is seen in a positive light. With this, if there is time availability in the lab schedule, it is advisible that it should be open for a variety of events and activities outside the scope of public engagement. IPIC will be utilizing an online booking system so that the wider community is able to book the space for training events or open days. In SSPC, the public engagement staff liaise with others who need to use the lab and engage with them about their requirements. Additionally, IPIC will have an interactive screen outside the lab so that employees and the public can see upcoming activities. Moreover, IPIC will have a dedicated lab manager who will manage relations with the rest of the institute including training and access.

Finally, certain university departments will also need to be aware of the lab. For example, SSPC has an agreement in place with the finance department as we often have to buy unusual equipment. Items such as sugar cubes and balloons are not catered for through the usual university contracts and we have spent time explaining our work and building relationships so that the approval of purchases is a smooth process.

## Case Study—Crystal Clear Project

To illustrate the benefits of a dedicated public engagement laboratory, a participatory SSPC project is outlined below. The *Crystal Clear* project is a codesign research project with a local girls secondary school (high school equivalent). Aged between 15 and 16, most of the girls are from socio-economically deprived backgrounds. The goal of the project is to employ real science research to engage and enthuse students with informal and nonformal educational practices. The *Crystal Clear* project asked students to grow piezoelectric crystals made of glycine and salt. Additionally, after 12 weeks of crystal growing and research, their results would be turned into a national citizen science project asking the public to grow their own crystals.

The initial codesign phase took place over 12 weeks in their school lab and our dedicated public engagement lab. Initially we visited the class to lay out the scope of the project and gain a common understanding of the task at hand. This was followed by a visit to our university and laboratory in which students were given a prescriptive way to grow crystals. Groups of students were also given iPads to record their work and research new ways of crystal growing. Depending on the progress of the students and the types of crystals being grown, we could strategically move between the school and SSPC lab depending on the work that was required for the following week. A dedicated lab space allowed for flexibility and a modular approach to the work. Furthermore, students had lengthy stays in the lab to develop their skills and become accustomed to the new setting.^10^

On a weekly basis, we would talk to students and teachers and redesign the crystal growing protocol. This would mean that we would have to source materials, potentially test them in our lab, and set them up for the next week. The lab, which is typically used for teaching and learning, turned into a more traditional lab, except that teenage researchers “owned” the space. Over the 12 weeks, the lab was needed to collate and curate hundreds of crystal growing attempts with diverse approaches including the use of different growing strata such as Petri-dishes, tapes, foil, 3D printed models, and silicon molds. Moreover, students tested initial ideas to aid crystal growing such as the addition of food coloring to more advanced ideas like the addition of powder activated charcoal. Furthermore, all of these crystals were grown in conditions where they would not be disturbed due to the lab having the sole purpose of public engagement. Through the gradual release of responsibility, students gained more autonomy and became more confident in their school lab and ours. They also understood the safety aspects in both laboratories and how more difficult tasks with higher levels of risk could take place in the SSPC lab.^12^

With this project, having a dedicated laboratory was pivotal. The space enabled us to host students in a way that suited their schedule and store vast amounts of equipment and experimental material that could not be disturbed and allowed for the researchers helping the students to host meetings and test new equipment set to be used on the next round of experiments. The lab removes administrative burdens^10^ and the need for constant cleaning and moving of equipment along with a host of benefits around bringing students onto campus to partake in real science.

The next phase of the *Crystal Clear* project is the expansion of the experimental designs codeveloped with the students to the larger citizen science venture. The lab will be used to store a large amount of equipment including chemicals like glycine and salt along with carboard mailers, beakers, and pipettes. Once collated, an assembly line will be set up in the lab to create kits to be distributed to the public. Initially predictions are that 200 kits will be required with the option to make more. Once distributed, the kits will be sent back directly to the lab.

## Conclusion

We hope that by outlining the key considerations and ideas pertaining to the design and implementation of a public engagement laboratory, members of the community will push for these types of spaces within their own institutions. A clear and logical approach to lab management can lead to a range of benefits to staff, participants, and your wider community.
